# Lipidomic Analysis of Liver Lipid Droplets after Chronic Alcohol Consumption with and without Betaine Supplementation

**DOI:** 10.3390/biology12030462

**Published:** 2023-03-16

**Authors:** Madan Kumar Arumugam, Sathish Kumar Perumal, Karuna Rasineni, Terrence M. Donohue, Natalia A. Osna, Kusum K. Kharbanda

**Affiliations:** 1 Research Service, Veterans Affairs Nebraska-Western Iowa Health Care System, Omaha, NE 68105, USA; 2 Department of Internal Medicine, University of Nebraska Medical Center, Omaha, NE 68198, USA; 3 Center for Molecular and Nanomedical Sciences, Sathyabama Institute of Science and Technology, Chennai 600119, India; 4 Department of Biochemistry & Molecular Biology, University of Nebraska Medical Center, Omaha, NE 68198, USA

**Keywords:** alcohol, betaine, steatosis, lipid droplets, lipidomics, cholesterol, triacylglycerol

## Abstract

**Simple Summary:**

Alcohol-associated liver disease is a major healthcare problem worldwide and is the third leading cause of preventable deaths in the US. Hepatic steatosis is the earliest manifestation of chronic alcohol misuse, characterized by accumulation of specialized fat storing organelles called lipid droplets (LDs). Our previous studies reported that the alcohol-induced increase in the number and size of LDs is attenuated by simultaneous treatment with the methyl group donor, betaine. In this study, we examined alcohol ± betaine-induced changes in the LD lipidome with respect to their size. Untargeted lipidomic analyses of the three different-sized hepatic LD fractions revealed higher phospholipids, cholesteryl esters, diacylglycerols, ceramides, and hexosylceramides in each fraction isolated from livers of ethanol-fed rats compared with the corresponding fractions of pair-fed controls. Betaine supplementation significantly attenuated the ethanol-induced LD lipidomic changes. We conclude that ethanol-induced changes in the hepatic LD lipidome may stabilize larger-sized LDs during steatosis development. Furthermore, betaine supplementation could effectively reduce the size and dynamics of LDs to attenuate alcohol-associated hepatic steatosis.

**Abstract:**

The earliest manifestation of alcohol-associated liver disease is hepatic steatosis, which is characterized by fat accumulation in specialized organelles called lipid droplets (LDs). Our previous studies reported that alcohol consumption elevates the numbers and sizes of LDs in hepatocytes, which is attenuated by simultaneous treatment with the methyl group donor, betaine. Here, we examined changes in the hepatic lipidome with respect to LD size and dynamics in male Wistar rats fed for 6 weeks with control or ethanol-containing liquid diets that were supplemented with or without 10 mg betaine/mL. At the time of sacrifice, three hepatic LD fractions, LD1 (large droplets), LD2 (medium-sized droplets), and LD3 (small droplets) were isolated from each rat. Untargeted lipidomic analyses revealed that each LD fraction of ethanol-fed rats had higher phospholipids, cholesteryl esters, diacylglycerols, ceramides, and hexosylceramides compared with the corresponding fractions of pair-fed controls. Interestingly, the ratio of phosphatidylcholine to phosphatidylethanolamine (the two most abundant phospholipids on the LD surface) was lower in LD1 fraction compared with LD3 fraction, irrespective of treatment; however, this ratio was significantly lower in ethanol LD fractions compared with their respective control fractions. Betaine supplementation significantly attenuated the ethanol-induced lipidomic changes. These were mainly associated with the regulation of LD surface phospholipids, ceramides, and glycerolipid metabolism in different-sized LD fractions. In conclusion, our results show that ethanol-induced changes in the hepatic LD lipidome likely stabilizes larger-sized LDs during steatosis development. Furthermore, betaine supplementation could effectively reduce the size and dynamics of LDs to attenuate alcohol-associated hepatic steatosis.

## 1. Introduction

Alcohol-associated liver disease (ALD) is a major healthcare problem and is the third leading cause of preventable deaths in the US [1,2,3,4]. Hepatic steatosis (fatty liver) is the earliest indicator of chronic alcohol misuse. It is characterized by lipid accumulation within specialized cytoplasmic organelles called lipid droplets (LDs) within hepatic parenchymal cells (hepatocytes). The pathogenic mechanism(s) for ethanol-induced hepatic lipid accumulation include enhanced uptake by hepatocytes of circulating fatty acids, accelerated intracellular fatty acid synthesis, and decelerated fatty acid oxidation and export [5,6,7]. Steatosis, the reversible stage of ALD, was once considered a benign condition. It is now regarded as the “first hit” that leaves the liver vulnerable to multiple subsequent hits, that enhance ALD progression to severe, irreversible stages [8]. Due to the close relationship between hepatic steatosis and progressive liver injury, steatosis is a prime target for therapeutic intervention. Therefore, lipidomic was employed here to examine whether it provides an early diagnosis of ALD for the eventual development of treatment options [9,10]. Recent analyses have revealed that chronic ethanol consumption causes a significant decline in hepatic phosphatidylcholine (PC) concomitant with the increase in fatty acids, diacylglycerols (DAG), and lysophosphatidylcholine in conjunction with an increase in unsaturated fatty acyl chains [11,12]. While these previously mentioned studies were all related to total hepatic lipidome, here, our objective was to specifically examine the LD lipidome and to quantify changes in these organelles during ethanol-induced steatosis development [13,14]. A key histopathological feature of steatosis is an increased LD number and size [15]. Based on these considerations, we hypothesized that, after ethanol exposure, the hepatic LD lipidome would vary with the size and dynamics of these lipid storing organelles. Here, we conducted an untargeted lipidomic analysis of LDs using electrospray ionization-tandem mass spectrometry of different-sized LD fractions isolated from livers of rats fed the control or ethanol liquid diet.

Our previous studies demonstrated that co-administration of betaine with ethanol prevented the development of alcohol-associated steatosis, as judged by a reduction in LD size and number compared with rats fed ethanol alone [15,16]. Therefore, in this study, our lipidomic analyses also included LD fractions isolated from livers of rats fed the betaine-supplemented ethanol diet.

## 2. Materials and Methods

### 2.1. Animal Experiments

Lieber-DeCarli control and ethanol liquid diets were purchased from Dyets Inc. (Bethlehem, PA, USA). Male Wistar rats, weighing from 180 to 200 g, were acquired from Charles River Laboratories, (Wilmington, MA, USA), weight-matched, adapted to liquid diet feeding, then pair-fed the Lieber-DeCarli control, ethanol, or the betaine-supplemented ethanol liquid diet [17] for 6 weeks, as described previously [16]. Briefly, the rats were divided into three groups of eight animals each. Group 1 was fed control diet, group 2 was fed ethanol diet, and group 3 was fed ethanol diet containing 1% (*w*/*v*) betaine. Each feeding day, rats in groups 1 and 2 were fed the same volume of diet consumed the previous day by rats in group 3. At the time of sacrifice, liver was excised and immediately processed for histology or for isolation of different-sized LD fractions. The remaining liver tissue was stored at −70 °C for subsequent analysis of total liver triacylglycerol (TAG), cholesterol, S-adenosylmethionine, and S-adenosylhomocysteine levels [15,16]. All animal care, use, and experimental procedures utilized for this study complied with the NIH guidelines and were approved by the Institutional Animal Care and Use Committee of the Omaha Veterans Affairs Medical Center.

### 2.2. Liver Histology

Formalin-fixed liver tissue was processed for hematoxylin-eosin staining and evaluated for lipid content [15].

### 2.3. Separation and Isolation of Different-Sized LDs

LDs of varying sizes were isolated from the liver tissues according to the protocol previously described [18,19]. Briefly, the excised liver was rinsed with ice-cold phosphate-buffered saline (PBS) and a 6 g portion was homogenized in 20 mM Tricine, (pH 7.8), containing 250 mM sucrose and 0.5 mM PMSF. Each homogenate was centrifuged at 500× *g* for 5 min at 4 °C to pellet tissue debris and blood cells. Each supernatant (8.5 mL) was transferred into an ultraclear SW 40 Ti ultracentrifuge tubes (Beckman #344059) and was overlaid with 3 mL of buffer B (20 mM HEPES, (pH 7.4), 100 mM KCl, and 2 mM MgCl_2_). Following centrifugation at 500× *g* for 20 min at 4 °C, the top white layer was collected into a 1.5-mL microcentrifuge tube and marked as “large-sized LDs” (LD1). Then, an equal volume of buffer B was added back (to replace the white buffy layer (just removed) and the gradient was recentrifuged at 2000× *g* for 20 min at 4 °C. The top white band was again collected into a 1.5-mL microcentrifuge tube, which was marked as “medium-sized LDs” (LD2). Then, another equal volume of buffer B was loaded onto the top and the gradient was recentrifuged at 8000× *g* for 20 min at 4 °C. The top white layer was again collected into a 1.5 mL-microcentrifuge tube, which was marked as smaller-sized LDs (LD3). Finally, each LD fraction collected was further purified by centrifuging it for 10 min at 4 °C at the g force that was originally used to acquire it. The underlying solution and pellet were removed, and the LDs were gently resuspended in buffer B. This final washing step was repeated until no pellet was visible after centrifugation.

### 2.4. Lipid Extraction

Total lipids were quantitatively extracted from pre-weighed liver sections and from each LD fraction, using a modified Folch lipid extraction method [20]. TAG and cholesterol levels were biochemically determined in the lipid extract of each liver and each LD fraction, as described [15,16].

### 2.5. BODIPY Staining of LDs

To assess LD size and purity, the different-sized LD fractions isolated from livers of rats fed control, ethanol, or betaine-supplemented ethanol diet were stained with 1,3,5,7-Tetramethyl-8-phenyl-4,4-difluoroboradiazaindacene (BODIPY 493/503; Invitrogen, Carlsbad, CA, USA), as previously described [21,22]. Briefly, 20 µL of each isolated LD fraction was placed on a slide and stained with 1 µg BODIPY 493/503 (Invitrogen, Carlsbad, CA, USA). All LD fractions were used undiluted, except for LD1 and LD2 fractions obtained from livers of ethanol-fed rats, which were diluted 1:20 and 1:4, respectively. After staining for 5 min, LDs were visualized under a Keyence BZ-X810 florescence microscope and images were captured. LD size was quantified using Keyence BZ-X810 Analyzer software [22].

### 2.6. Mass Spectrometry Analysis

An automated electrospray ionization mass spectrometry (ESI-MS/MS) approach was used for lipidomic analysis. Data acquisition and analyses were carried out at the Kansas Lipidomics Research Center (Manhattan, KS, USA) as described previously [23,24].

### 2.7. Statistical Analysis

Data were analyzed by one-way ANOVA, followed by Tukey’s post-hoc unpaired test for comparisons among groups. Comparisons with *p*-values ≤ 0.05 were considered statistically significant.

## 3. Results

### 3.1. Body Weights, Liver Weights, and Liver Histology

**Figure 1:**
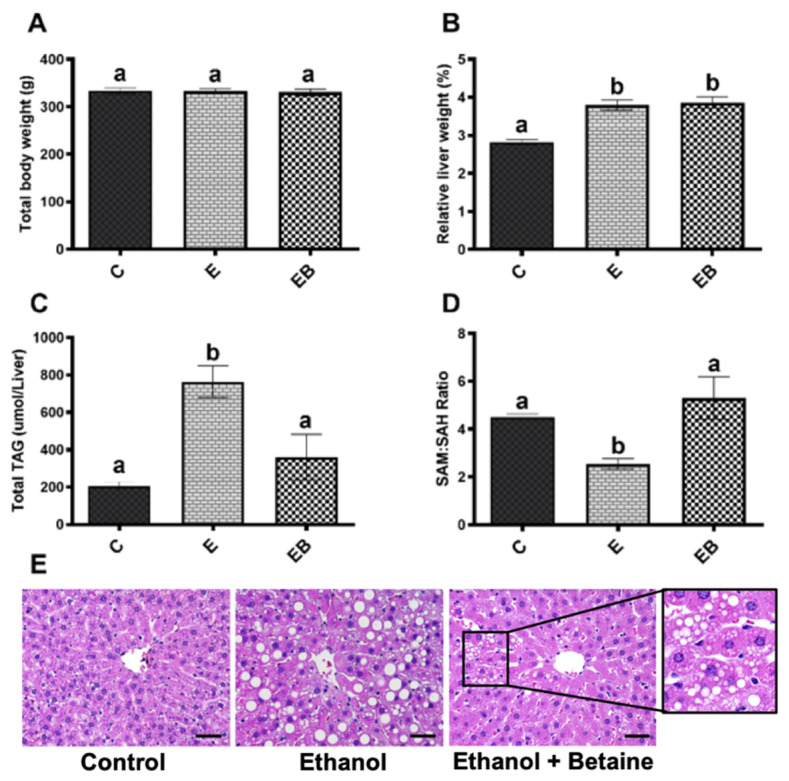
(**A**) Body weight, (**B**) relative weight of liver, total hepatic (**C**) triacylglycerol (TAG), and (**D**) S-adenosylmethionine (SAM):S-adenosylhomocysteine (SAH) ratio of rats fed control (C), ethanol (E), or betaine-supplemented ethanol (EB) diet. (**E**) Photomicrograph of representative H&E- stained liver sections of control, ethanol, and betaine-supplemented ethanol-diet fed rats. Scale bar = 100 µm. Micro- and macrovesicular steatosis was observed in liver sections of ethanol-fed rats, while liver sections of betaine-supplemented ethanol-diet fed rats showed microvesicular steatosis (inset: Magnified image). Data are presented as the mean ± SEM (*n* = 8); values not sharing a common letter differ significantly from each other at *p* ≤ 0.05.

The final body weights and relative liver weights of control and experimental rats are shown (Figure 1A,B, respectively). There were no differences in the final body weights among the three animal groups (Figure 1A). Compared with pair-fed controls, absolute liver weight (in grams) and relative liver weight (in grams/100 g BW) were significantly higher in both ethanol-fed rats and in betaine-supplemented ethanol-fed rats (Figure 1B). The observed hepatic TAG levels (Figure 1C), SAM:SAH ratio (Figure 1D), and liver histopathological evaluations (Figure 1E) were consistent with our previously published results [15,16]. H&E-stained liver sections of ethanol-fed rats exhibited cytoplasmic vacuolization, with micro- and macrovesicular steatosis (Figure 1E). However, some evidence of microvesicular steatosis was evident in livers of rats fed the betaine-supplemented ethanol diet at higher magnification (Figure 1E).

### 3.2. Effect of Ethanol and Betaine Co-Administration on LD Sizes

**Figure 2:**
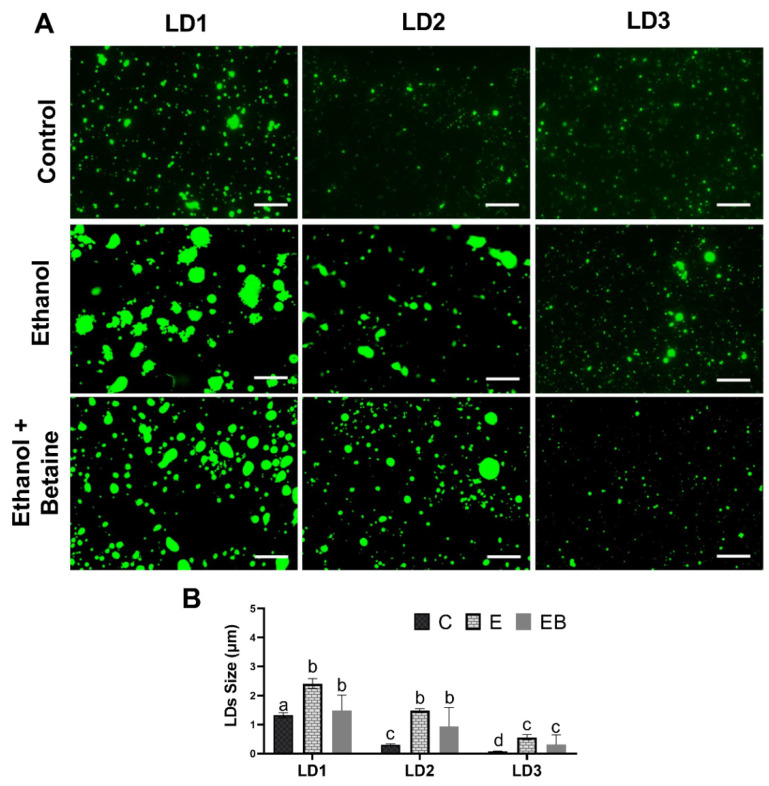
(**A**) Characterization determined by BODIPY staining of different fractions of liver LDs (LD1-LD3) of rats fed control (C), ethanol (E), or betaine-supplemented ethanol (EB) diet. Scale bar = 50 µm. (**B**) LD size measurements in the different LD fractions. Data are presented as the mean ± SEM (*n* = 8); values not sharing a common letter significantly differ from each other at *p* ≤ 0.05. Note that all LD fractions were stained undiluted, except for LD1 and LD2 fractions obtained from livers of ethanol-fed rats, which were diluted 1:20 and 1:4, respectively.

BODIPY 493/503 is a green lipophilic fluorescent dye that allows microscopic detection of LDs [25]. Quantification of BODIPY-stained LD isolated from livers of rats fed control, ethanol, or betaine-supplemented ethanol diet still revealed some heterogeneity in LD sizes in each LD fraction. We confirmed the sizes of isolated LDs by BODIPY 493/503 staining (Figure 2). Generally, the LD1 fraction comprised of large-sized LDs (LD1) followed by medium-sized LDs (LD2) and smaller-sized LDs (LD3). LD fractions (LD1-LD3) isolated from rats fed the control or the betaine-supplemented ethanol diet exhibited generally reduced sizes compared with the corresponding fractions of ethanol treated rats as shown in Figure 2.

### 3.3. Effect of Ethanol and Betaine Co-Administration on TAG and Total Cholesterol Levels in LD Fractions

**Figure 3:**
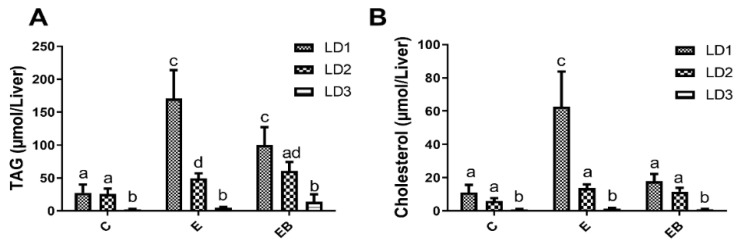
(**A**) Triacylglycerol (TAG) and (**B**) total cholesterol levels in different fractions of liver LDs (LD1-LD3) of rats fed (C), ethanol (E), or betaine-supplemented ethanol (EB) diet. Data are presented as the mean ± SEM (*n* = 8); values not sharing a common letter significantly differ from each other at *p* ≤ 0.05.

LDs store fatty acids in the form of neutral lipids, including TAG and cholesteryl esters [26]. The lipid extracts from each LD fraction were quantified, which revealed significantly higher TAGs and cholesterol in LD fractions of ethanol-fed rats compared with the corresponding fractions of pair-fed controls (Figure 3A,B). Inclusion of betaine in the ethanol diet effectively attenuated the TAG and cholesterol levels in each LD fraction compared with those levels in the fractionated LDs of rats fed ethanol alone (Figure 3A,B). This attenuation of the lipid contents in LDs is consistent with our previously published findings of total TAG in livers of rats fed the betaine-supplemented ethanol diet [15,16].

### 3.4. Lipidomic Analyses of Different Liver LD Fractions

We performed lipidomic analyses on the individual lipid classes of distinct phenotypes in different-sized LDs from our three groups of animals. A total of 21 lipid classes, constituting 1914 different lipid species were identified and analyzed from LD extracts of large, medium, and smaller-sized LDs isolated from livers of rats fed control, ethanol, and betaine-supplemented ethanol diets. This lipidomic approach permitted the quantitative analysis of phospholipids (PL), ceramides (Cer), hexosylceramides (HexCer), cholesteryl esters (CE), diacylglycerol (DAG), TAG, and glycerophospholipids, including PC, phosphatidylethanolamine (PE), phosphatidylserine (PS), and phosphatidylinositol (PI) species, which were characterized by their carbon chain lengths and the numbers of double bonds in their constituent acyl residues.

#### 3.4.1. Effect of Ethanol and Betaine Co-Administration on LD1

**Figure 4:**
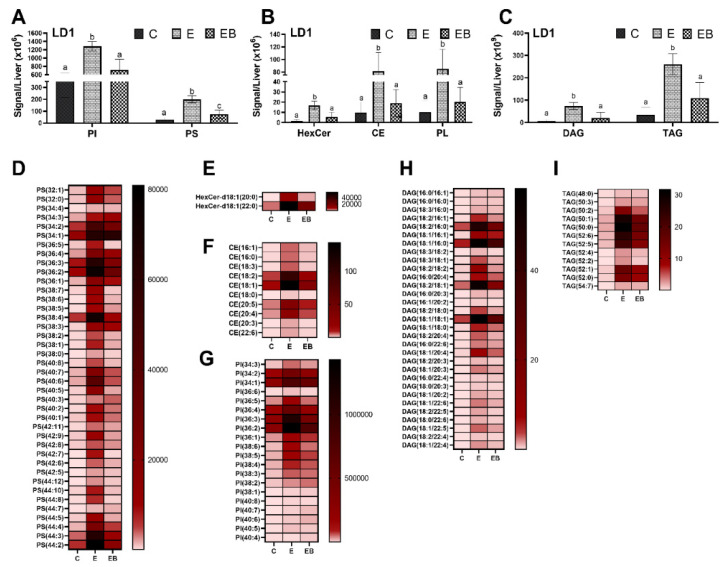
Effects of ethanol and betaine on the lipid species in LD1 fraction isolated from livers of rats fed control (C), ethanol (E), or betaine-supplemented ethanol (EB) diet. (**A**) Phosphatidylinositol (PI) and phosphatidylserine (PS). (**B**) Hexosylceramides (HexCer), cholesteryl esters (CE), and phospholipids (PL). (**C**) Diacylglycerols (DAG) and triacylglycerol (TAG) lipid species identified in LD1 were plotted according to the signal per liver. Data are presented as the mean ± SEM (*n* = 8); values not sharing a common letter significantly differ from each other at *p* ≤ 0.05. Panels (**D**–**I**) are data presented as heatmap showing the relative distribution of each lipid species in LD1. Each row represents the normalized intensities of a unique chromatographic feature. The features are color coded by row with dark brown indicating high intensity, brown indicating low intensity, and light pink indicating below limit of detection.

The level of PI, PS, HexCer, CE, PL, DAG, and TAG lipid species in the larger LDs (LD1) from livers of control, ethanol, and betaine-supplemented ethanol-fed rats are shown in Figure 4. All these lipid species were significantly elevated in LD1 from ethanol-fed rats compared with pair-fed control rats. Betaine co-treatment significantly decreased the aforementioned lipid species (Figure 4A–C). We analyzed the total PI (Figure 4A) and 20 distinct PI variants (Figure 4G) in ethanol LD1 that were higher than those in control LD1. Pl is a major source of arachidonic acid, a precursor of the proinflammatory eicosanoid and an early indicator of steatosis [27,28]. In this study, we found 4.9-fold (*p* < 0.05) higher PI (36:3) 1-linoleoyl-2-oleoyl-sn-glycero-3-phosphoinositol and 4.8-fold (*p* < 0.05) higher PI (36:2) 1-linoleoyl-2-stearoyl-sn-glycero-3-phosphoinositol in ethanol LD1 compared with control LD1 (Table S1). Alterations in the phospholipid compositions of liver cell membranes occur during ALD pathogenesis [29]. Interestingly, we observed 10.3-fold higher phosphatidylserine (PS) levels in ethanol LD1 (*p* < 0.001) compared with control LD1, which was decreased by 2.7-fold with (*p* < 0.05) by betaine co-treatment (Figure 4A). Additionally, the heat map generated revealed significantly higher levels of 36 different PS species in LD1 fraction of ethanol-fed rats when compared with control LD1 (Figure 4D; *p* < 0.05). Specifically, as shown in Table S2, PS (32:0) 1,2-dihexadecanoyl-sn-glycero-3-phosphoserine was 11.7-fold, PS (34:2) 1-dodecanoyl-2-(13Z,16Z-docosadienoyl)-glycero-3-phosphoserine was 8.8-fold, and PS (38:4) 1-(9Z-octadecenoyl)-2-(8Z,11Z,14Z eicosatrienoyl)-glycero-3-phosphoserine level was 13.2-fold higher in ethanol compared with those from controls (*p* < 0.05). This ethanol-induced increase was effectively reduced after betaine co-treatment. Interestingly, compared with the corresponding fractions from livers of ethanol-fed rats, PS (34:4), PS (36:4), PS (40:3), PS (42:8), and PS (44:7) were unaltered in larger-sized LDs by betaine treatment.

Similarly, other lipid subpopulations, such as HexCer (18:1/20:0) N-(eicosanoyl)-sphing-4-enine and HexCer (18:1/22:0) N-(docosanoyl)-sphing-4-enine were elevated by 18.8-fold (*p* < 0.05) and 15-fold (*p* < 0.05), respectively, in LD1 fractions of ethanol-fed rats compared with those of pair-fed controls. The levels of these HexCer were significantly lowered by betaine co-treatment (Figure 4B,E; Table S3).

Cholesteryl esters (CE) are long-chain fatty acids linked to the hydroxyl group of cholesterol. They are significantly less polar molecules than free cholesterol and appear to be the preferred molecular form for transport in plasma and storage in liver [30]. We found 10 distinct CEs that were significantly elevated in ethanol LD1, particularly CE (18:2) cholesteryl linoleate and CE (20:3) cholesteryl eicosadienoic acid by 13.4- and 14.8-fold, respectively, over control LD1. Compared with ethanol LD1, the levels of these CEs were significantly lower in LD1 of betaine co-treated animals (Figure 4F; Table S3).

Similarly, 31 different DAGs were significantly elevated in the ethanol LD1 fraction compared with the control LD1 (Figure 4H). Specifically, DAG (18:2/18:2) glyceryl 1,2-dilinoleate was 19.8-fold (*p* < 0.05) and DAG (18:2/18:1) 1-linoleoyl-2-vaccenoyl-sn-glycerol was 19.7-fold (*p* < 0.01) higher in ethanol LD1 compared with control LD1. These DAG species were significantly decreased in the LD1 fraction of rats fed the betaine-supplemented ethanol diet (Figure 4H; Table S3). In addition, 12 different TAGs were significantly elevated in the ethanol LD1 fraction compared with the control LD1 (Figure 4I; Table S4). Specifically, TAG (50:2), TAG (50:1), TAG (50:0), TAG (52:6), TAG (52:5), TAG (52:2), TAG (52:1), and TAG (52:0) were higher in ethanol LD1 compared with control LD1. As seen with other lipid classes, betaine co-treatment with ethanol significantly reduced the levels of these TAG species in LD1 fractions compared with the corresponding fraction from ethanol-fed rat livers.

#### 3.4.2. Effect of Ethanol and Betaine Co-Administration on LD2

**Figure 5:**
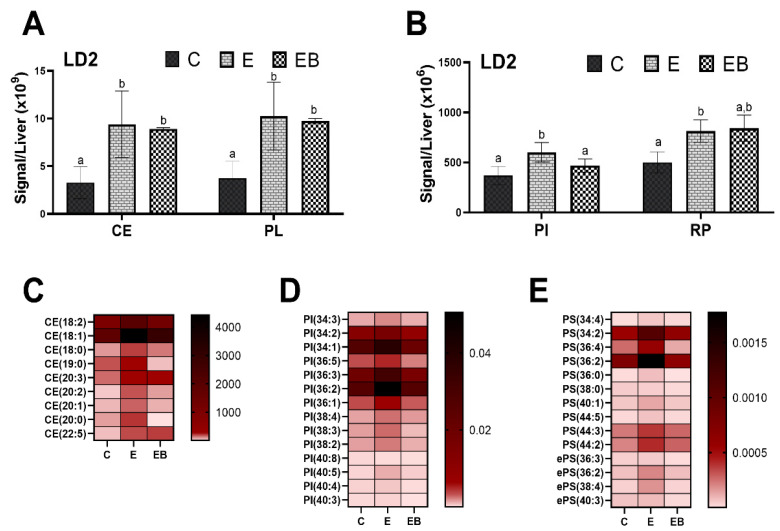
Effects of ethanol and betaine on the lipid species in liver LD2 fraction of rats fed control (C), ethanol (E), or betaine-supplemented ethanol (EB) diet. Respective lipid species. (**A**) Cholesteryl esters (CE) and phospholipids (PL). (**B**) Phosphatidylinositol (PI) and common phospholipids (RP) of LD2 are identified and plotted according to the signal per liver. Data are presented as the mean ± SEM (*n* = 8); values not sharing a common letter differ significantly from each other at *p* ≤ 0.05. (**C**–**E**) Data in the heatmap show the level of each sub-populated lipid species from LD2. Each row represents the normalized intensities of a unique chromatographic feature. The features are color-coded by row with dark brown indicating high intensity, brown indicating low intensity, and lightpink indicating below limit of detection.

We further investigated the quantitative changes in medium-sized LDs (LD2). Similar to LD1, we found that the lipid species CE, PL, PI, as well as more common phospholipids (RP) were again, significantly higher in LD2 fractions from livers of ethanol-fed rats compared with their pair-fed control rats (Figure 5A,B). However, the majority of other abundant lipid species, including PC, PE, DAG, and TAG were essentially equal in the control and ethanol LD2. The heat map shows the subclass of a total of nine different CE species (Figure 5C), of which five exhibited no changes with ethanol alone or with betaine co-treatment. However, other CEs, such as CE (18:2) cholesteryl linoleic acid, CE figure (18:1) cholesteryl oleate, CE (18:3) 1-g-linolenoyl-cholesterol, CE (16:0) cholesterol palmitate, and CE (18:3) 1-a-linolenoyl-cholesterol were significantly higher in ethanol LD2 fraction compared with control LD2, which were all significantly reduced in the corresponding fraction of rats fed the betaine-supplemented ethanol diet (Table S5). Similarly, other 14 different glycerophospholipids of PI and PS were analyzed as shown in Figure 5D,E, respectively. The fold changes, as shown in Table S6, demonstrate that PI (34:3), PI (34:1), PI (36:5), PI (36:3), PI (36:2), PI (36:1), PI (38:4), PI (38:3), PI (38:2), and PI (40:5) were significantly increased in ethanol LD2 fraction compared with control LD2. Other PI species, such as PI (34:2), PI (40:8), PI (40:4), and PI (40:3) are unaltered between the LD2 of control and experimental groups. Similarly, several PS species were significantly increased in ethanol LD2 compared with control LD2, as shown (Table S7), which were reduced by betaine co-treatment. Other species of PS, such as PS (34:4), PS (38:0), PS (44:5), PS (36:3), and PS (40:4) were unaltered between the LD2 fractions obtained from the different experimental groups.

#### 3.4.3. Effect of Ethanol and Betaine Co-Administration on LD3

**Figure 6:**
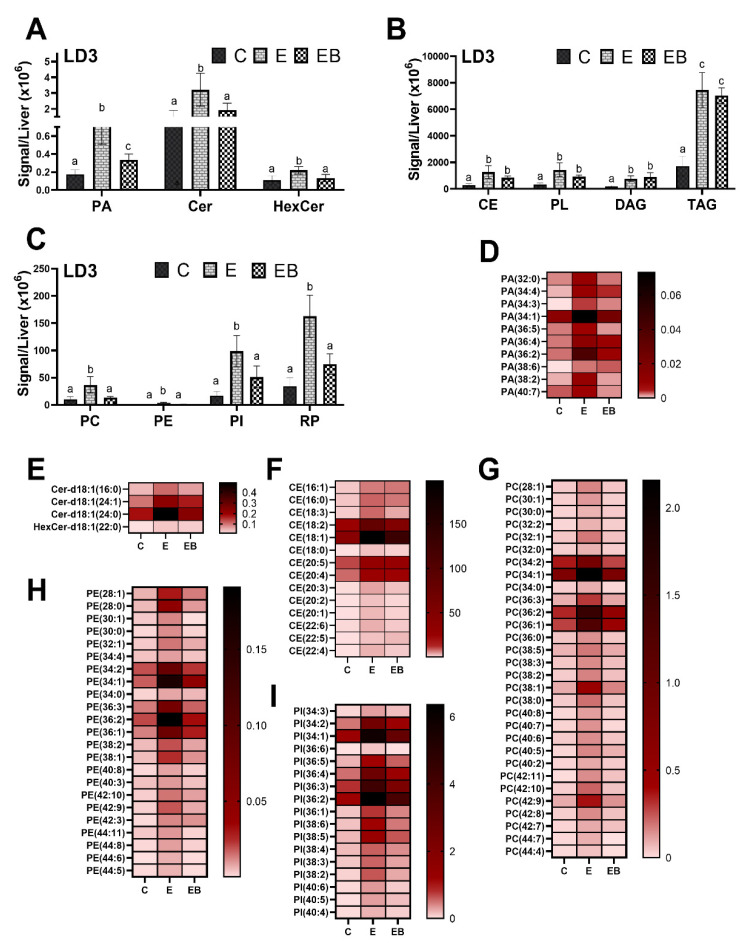
Effects of ethanol and betaine on the lipid species in LD3 fractions from livers of rats fed control (C), ethanol (E), or betaine-supplemented ethanol (EB) diet. Respective lipid classes in LD3 are identified and plotted according to the signal per liver. (**A**) Phosphatidic acid (PA), ceramides (Cer), and hexosylceramides (HexCer). (**B**) Cholesteryl esters (CE), phospholipids (PL), diacylglycerols (DAG), and triacylglycerol (TAG). (**C**) Phosphatidylcholine (PC), phosphatidylethanolamine (PE), phosphatidylinositol (PI), and common phospholipids (RP). Data are presented as the mean ± SEM (*n* = 8); values not sharing a common letter significantly differ from each other at *p* ≤ 0.05. (**D**–**I**) Data in the heatmap showed the level of each sub-populated lipid species from LD3. Each row represents the normalized intensities of a unique chromatographic feature. The features are color-coded by row with dark brown indicating high intensity, brown indicating low intensity, and light pink indicating below limit of detection.

As performed previously for the other LD fractions, we examined the lipidomes of smaller-sized LDs (LD3) from livers of rats fed control, ethanol, or betaine-supplemented ethanol diet. The majority of the abundant lipid species, such as phosphatidic acid (PA), Cer, HexCer, CE, PL, DAG, TAG, PC, PE, PI, and RP were significantly higher in ethanol LD3 compared with control LD3 (Figure 6A–C). Additionally, the heat map showed the level of glycerophospholipid class, of which a total of 10 different PA species (Figure 6D), such as PA (32:0) 1-arachidoyl-2-lauroyl-sn-phosphatidic acid, PA (34:1) 1-palmitoyl-2-vaccenoyl-sn-glycero-3-phosphate, PA (36:5) 1-stearidonoyl-2-oleoyl-sn-phosphatidic acid, PA (38:2) 1-nervonoyl-2-myristoleoyl-sn-phosphatidic acid, and PA (40:7) 1-meadoyl-2-arachidonoyl-sn-phosphatidic acid were significantly higher in ethanol LD3 compared with control LD3 (Table S8). Other PA species, such as PA (34:4), PA (36:4), PA (36:2), and PA (38:6) were unaltered in these LD3.

Ceramides are biologically active sphingolipids that act as critical factors in the pathogenesis of ALD [31] Here, we observed higher levels of three distinct Cer, i.e., Cer (18:1/16:0), Cer (18:1/24:1), and Cer (18:1/24:0) in ethanol LD3 compared with control LD3. After betaine co-treatment, these Cer levels were significantly lower than in LD3s from ethanol-fed rats (Figure 6E; Table S8). One HexCer 18:1 (22:0) (Figure 6E), 14 different CE (Figure 6F; Table S8), 30 different PC (Figure 6G; Table S9), 23 different PE (Figure 6H; Table S10), and 17 different PI (Figure 6I; Table S11) were also increased in ethanol LD3 fractions compared with control LD3 fractions. Rats fed the betaine-supplemented ethanol diet exhibited significantly lower levels of these species in the corresponding fraction from ethanol-fed rats. As shown, the total DAG and TAG were 3.7-fold (*p* < 0.001) and 3.8-fold higher (*p* < 0.05), respectively, in ethanol LD3 compared with the corresponding fraction of pair-fed controls (Figure 6B). However, while these total DAG and TAG levels were unaltered by the betaine co-treatment, there was a marked decrease in mostly mono- and poly-unsaturated fatty acid species in LD3 fractions.

### 3.5. Analysis of PC:PE Ratio

**Figure 7:**
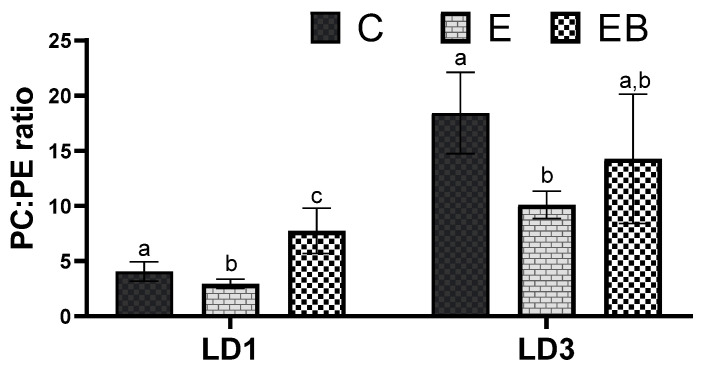
Analysis of PC:PE ratio in larger- (LD1) and smaller-sized LDs (LD3) of rats fed control (C), ethanol (E), or betaine-supplemented ethanol (EB) diet. Data are presented as the mean ± SEM (*n* = 8); values not sharing a common letter significantly differ from each other at *p* ≤ 0.05.

Interestingly, the larger-sized fraction, LD1, had significantly lower PC:PE ratio than the smaller-sized LD3 fraction, irrespective of their treatments (Figure 7). When we compared the ratios between treatments, we observed that the PC:PE ratio was significantly lower (by 1.2-fold) in ethanol LD1 (and LD3 by 2.1-fold) compared with their respective controls, LD1 and LD3 (*p* < 0.05). The PC:PE ratio in both the largest LDs (LD1) and smallest LDs (LD3) reverted to near normal levels after the betaine treatment.

## 4. Discussion

Hepatic steatosis, the first pathological sign of liver disease in the ALD spectrum, is characterized by enhanced production and slower catabolism of LDs in hepatocytes [32], as well as the expansion of LD size [15,33,34]. Here, we sought to ascertain how chronic ethanol administration alters the liver LD lipidome in Wistar rats and whether these lipidomic changes were prevented or reversed by betaine that was simultaneously co-administered with ethanol. Previous studies reported that there are significant lipidomic changes in livers of ethanol-fed animals [11,35,36]. We recently reported that chronic alcohol consumption increases the number and size of liver LDs [15,32]. Our further studies indicated that the accumulation of large-sized LDs in livers of ethanol-fed rats likely occurs via increased generation of smaller-sized LDs and their subsequent fusion and that the generated larger-sized LDs accumulate due to decreased lipolysis of their lipid stores [22]. The current study was conducted to explore whether changes in the lipid LD profile after chronic alcohol administration is related to the size of the LDs, which, in turn, controls their dynamics, as shown previously [22]. Our other objective was to determine whether betaine treatment prevented these LD lipidome alterations. Here, we extracted three different-sized LDs from livers of control, ethanol, and betaine-supplemented ethanol-fed rats and performed lipidomic analyses.

Our untargeted lipidomic analysis revealed that ethanol administration significantly altered several hepatic lipids, including phospholipids and glycerophospholipids in different-sized LDs. Cer and HexCer belong to the group of cerebrosides within the sphingolipids. They are key precursors for the biosynthesis of dihexosylceramides, which are key structural components of intracellular membranes and lipid rafts [37,38]. These ceramides act as a bioactive lipid that can impair insulin signaling, induce oxidant stress, impair fatty acid oxidation, and enhance lipoprotein aggregation, all of which are linked to ALD pathogenesis [39]. Our results are consistent with the results reported earlier, documenting that chronic alcohol consumption significantly elevates specific Cer in the liver, while it reduces Cer plasma levels [35]. We observed higher levels of sphingolipids, such as 2.7-fold increase in Cer C18:1 (24:0), 18.8-fold increase in HexCer -C18:1 (20:0), and 15-fold increase in HexCer C18:1 (22:0) in each fraction of LDs from livers of ethanol-fed rats. Since these sphingolipids are the major source of membranes [40] and larger sized LDs are seen in livers of ethanol-fed rats [15,32], whether this increase may be regulating the LD size or vice versa is not known. There is, however, a definite link between the LD size and this lipid class, since the ethanol-induced increase in sphingolipids was significantly attenuated after the betaine co-treatment, which coincided with a reduction in LD size. Furthermore, the mechanistic link between hepatoprotective effect of betaine and sphingolipid level is not completely clear. Since sphingolipids have cell signaling properties that trigger inflammation, apoptosis, and insulin resistance [41,42], the reduction in their levels by betaine treatment could be related to the attenuation in various insults, (including alcohol)-induced increase in inflammation, apoptosis, and insulin resistance [43,44,45,46].

Studies have reported that chronic alcohol consumption promotes accumulation of neutral lipids in LDs within hepatocytes [11,47,48]. These neutral lipids, such as CE and TAG are uncharged, have no signaling properties, and are often enclosed within LDs [13,49,50]. Increased hepatic accumulation of these neutral lipids is a characteristic feature of alcohol-associated steatosis as shown by other lipidomic studies [35,50,51]. Here, we observed similar increases, with notable elevations in 16- and 18-carbon fatty acids, such as C (16:1), C (18:0), C (18:3) along with eicosatrienoic acid C (20:3) and docosahexaenoic acid C (22:6) in LD fractions of ethanol-fed rats compared with control counterparts. Biochemical analyses corroborated the increase in cholesterol and TAG in each LD fraction from alcohol-fed rats compared with rats fed the control or the betaine-supplemented ethanol diet.

Alcohol consumptions drive hepatic TAG synthesis via enhanced de novo fatty acid synthesis and increased uptake of adipose lipolysis-derived circulating fatty acids [52]. These alcohol-induced changes, i.e., increased de novo lipogenesis in hepatocytes and the increase in adipose lipolysis are a consequence of reduced SAM:SAH ratio [21,22]. Another factor for alcohol-induced increased TAG accumulation in hepatocytes is a reduction in very-low density lipoprotein (VLDL) secretion [53], which is a major pathway for exporting fat and preventing hepatic steatosis development. VLDL biogenesis is regulated by the availability of TAG stored in LDs, which must be hydrolyzed to provide a substrate for VLDL assembly and subsequent secretion [54,55,56,57]. Up to 70% of the TAGs packaged and secreted by hepatocytes in VLDLs are derived via lipolysis of LD TAG stores [57]. Indeed, we previously reported that hepatocytes isolated from ethanol-fed rats display a decrease in the rate of lipolysis compared with controls [22,32]. Subsequent studies from our laboratory showed that the reduced lipolysis is due to the lower SAM:SAH ratio, which reduces the activation of important lipases that target LD TAG stores [22]. In this study, several distinct DAG and TAG species were detected in each LD fraction, with significantly increased levels observed in those isolated from livers of alcohol-fed rats compared with controls. We believe that these increased levels result from the reduced hydrolysis of LD TAG stores [32], favoring DAG/TAG accumulation over their catabolism to fatty acids, which must be shuttled to mitochondria (for β-oxidation) or to the endoplasmic reticulum for re-esterification to form the VLDL core. The reduction in TAG/DAG levels after betaine treatment indicates that betaine, by normalizing the SAM:SAH ratio, must promote lipolysis of LD TAG stores, as the alcohol-induced impairment of VLDL synthesis and secretion was restored to normal after this treatment [53].

Phosphatidylcholine (PC) is a critical component of cell membranes and a central player in lipid metabolism [58,59]. PC in the liver is synthesized from choline via the CDP-choline pathway or by methylation of PE via phosphatidylethanolamine methyltransferase (PEMT)-mediated catalysis [60]. Importantly, studies have shown that a decline in the PC:PE ratio correlates with a decreased hepatocyte membrane potential [61]. The latter change in membrane integrity leads to hepatocyte damage or lysis and inflammation, promoting the progression of steatosis to steatohepatitis [61,62]. Limited studies have investigated the phospholipid composition and reported a reduction in PC levels in livers of ethanol-fed rats [11]. Even fewer studies have examined the changes in LDs, despite PC being the most abundant phospholipid (followed by PE) in the LD monolayer, which shields the neutral lipid core from the surrounding cytosol [24,63]. Interestingly, previous studies implicated that the PC:PE ratio regulates the size of LDs, which, in turn, influences the access of lipases to the LD TAG stores [64,65]. Others have also confirmed that the relative abundance of PC and PE regulate the sizes and dynamics of LDs [66]. Indeed, we observed a lower PC:PE ratio in total LDs isolated from livers of ethanol-fed rats compared with controls [32]. We further showed that the reduction in PC:PE ratio can lead to the over-abundance of large LDs through selective recruitment of class II anti-lipolytic LD proteins [32,67]. In line with these observations, here, we observed that larger-sized LDs (LD1) had a much lower PC:PE ratio than the smaller-sized LD3, irrespective of the treatment. Furthermore, this ratio was significantly lower in LD fractions from ethanol-fed rats compared with their pair-fed controls. This altered ratio returned to near normal after betaine treatment. Restoration of the PC:PE ratio in LD sub-fractions by betaine co-treatment coincided with the significant attenuation of ethanol-induced hepatic steatosis. It is possible that these PC:PE ratio changes in LDs are related to changes in the PEMT-catalyzed reaction to generate PC. We have shown that the PEMT-mediated catalysis is modulated by hepatocellular SAM:SAH ratio, and therefore is impaired in livers of ethanol-fed rats and normalized by betaine-supplementation [16,34]. We are currently conducting proteomic studies to determine whether the levels/activity of enzymes of the CDP-choline/PEMT pathway are altered in the different-sized fractions.

## 5. Conclusions

In conclusion, our findings indicate that ethanol-induced changes in the LD lipidome, especially changes in the PC:PE ratio, likely stabilizes larger-sized LD fractions during fatty liver development. The increased size of LDs may also be responsible for the reduced lipolysis of the TAG stores, causing their accumulation in the hepatocytes. Betaine supplementation could effectively alter LD structure to reduce LD sizes, thereby accelerating LD turnover to attenuate or prevent alcohol-induced steatosis.

## Data Availability

The data generated or analyzed during this study are included in this published article and its supplementary files.

## Supplementary Materials

The following supporting information can be downloaded at: https://www.mdpi.com/article/10.3390/biology12030462/s1, Table S1: The differences in the phosphatidylinositol (PI) lipidome of LD1 isolated from the livers of rats fed control (C), ethanol (E) or betaine-supplemented ethanol (EB) diet; Table S2: The differences in the phosphatidylserine (PS) lipidome of LD1 isolated from the livers of rats fed control (C), ethanol (E) or betaine-supplemented ethanol (EB) diet; Table S3: The differences in the hexosylceramide (HexCer), cholesteryl esters (CE) and diacylglycerol (DAG) lipidome of LD1 isolated from the livers of rats fed control (C), ethanol (E) or betaine-supplemented ethanol (EB) diet; Table S4: The differences in the triacylglycerol (TAG) lipidome of LD1 isolated from the livers of rats fed control (C), ethanol (E) or betaine-supplemented ethanol (EB) diet; Table S5: The differences in the cholesteryl esters (CE) lipidome of LD2 isolated from the livers of rats fed control (C), ethanol (E) or betaine-supplemented ethanol (EB) diet; Table S6: The differences in the phosphatidylinositol (PI) lipidome of LD2 isolated from the livers of rats fed control (C), ethanol (E) or betaine-supplemented ethanol (EB) diet; Table S7: The differences in the phosphatidylserine (PS) and ePS lipidome of LD2 isolated from the livers of rats fed control (C), ethanol (E) or betaine-supplemented ethanol (EB) diet; Table S8: The differences in the phosphatidic acid (PA), Ceramides and cholesteryl esters (CE) lipidome of LD3 isolated from the livers of rats fed control (C), ethanol (E) or betaine-supplemented ethanol (EB) diet; Table S9: The differences in the phosphatidylcholine (PC) lipidome of LD3 isolated from the livers of rats fed control (C), ethanol (E) or betaine-supplemented ethanol (EB) diet; Table S10: The differences in the phosphatidylinositol (PI) lipidome of LD3 isolated from the livers of rats fed control (C), ethanol (E) or betaine-supplemented ethanol (EB) diet; Table S11: The differences in the phosphatidylethanolamine (PE) lipidome of LD3 isolated from the livers of rats fed control (C), ethanol (E) or betaine-supplemented ethanol (EB) diet.
