# Supplementary material for: Lipidomic Analysis of Liver Lipid Droplets after Chronic Alcohol Consumption with and without Betaine Supplementation
Source: Biology (Basel). 2023 Mar 16;12(3):462. doi: 10.3390/biology12030462 (PMC10045066; doi:10.3390/biology12030462)
Supplement: Supplementary file 1 [file biology-12-00462-s001.zip › biology-1985201-supplementary.pdf]

biology

MDPI

Supplementary Material

Lipidomic analysis of Liver Lipid Droplets after Chronic Alcohol Consumption with and without Betaine Supplementation

Madan Kumar Arumugam [1,2,3] Sathish Kumar Perumal [1,2] Karuna Rasineni [1,2] Terrence M. Donohue Jr. [1,2,4] Natalia A. Osna [1,2] and Kusum K. Kharbanda [1,2,4,*]

Supplementary Tables

Table S1. The differences in the phosphatidylinositol (PI) lipidome of LD1 isolated from the livers of rats fed control (C), ethanol (E) or betaine-supplemented ethanol (EB) diet.

Lipid Species	C vs E		E vs EB		C vs EB	
	Fold change ↑	*p*-value	Fold change↓	*p*-value	Fold change ↑	*p*-value
PI(34:3)	3.6	0.003376	1.6	0.0327	2.2	0.075
PI(34:2)	3.4	0.000773	1.9	0.0034	1.8	0.088
PI(34:1)	3.0	0.000214	1.3	0.021456	2.2	0.003109
PI(36:6)	3.7	0.015325	2.2	0.060907	1.7	0.18477
PI(36:5)	3.1	0.000876	2.3	0.004074	1.3	0.313934
PI(36:4)	4.1	0.00028	2.8	0.000651	1.5	0.246736
PI(36:3)	4.9	0.000915	2.6	0.004527	1.9	0.111229
PI(36:2)	4.8	0.000668	1.9	0.009038	2.6	0.007531
PI(36:1)	3.4	0.000842	1.5	0.03806	2.3	0.014817
PI(38:6)	6.7	0.003004	2.3	0.038894	3.0	0.069835
PI(38:5)	5.1	0.003438	1.5	0.136593	3.4	0.05665
PI(38:4)	4.0	0.010103	1.4	0.221509	2.9	0.047197
PI(38:3)	3.9	0.016944	1.2	0.341466	3.4	0.001943
PI(38:2)	2.7	0.022299	0.7	0.092458	3.7	2.48E-05
PI(38:1)	4.4	0.10391	0.8	0.359323	5.5	0.003894
PI(40:8)	4.0	0.001911	1.2	0.097239	3.2	0.001665
PI(40:7)	7.9	0.008544	0.5	0.10375	16.1	0.01588
PI(40:6)	3.6	0.018457	0.5	0.115726	6.8	0.021355
PI(40:5)	4.8	0.019778	1.3	0.288662	3.6	0.010745
PI(40:4)	1.0	0.493	0.3	0.000	3.2	0.010

Table S2. The differences in the phosphatidylserine (PS) lipidome of LD1 isolated from the livers of rats fed control (C), ethanol (E) or betaine-supplemented ethanol (EB) diet.

Lipid Species	C vs E		E vs EB		C vs EB	
	Fold change ↑	p-value	Fold change↓	p-value	Fold change ↑	p-value
PS(32:1)	8.8	0.026	2.1	0.095	4.2	0.095
PS(32:0)	11.7	0.025	3.0	0.078	4.0	0.078
PS(34:4)	6.3	0.015	1.1	0.047	5.8	0.047
PS(34:3)	3.5	0.015	0.7	0.032	5.0	0.032
PS(34:2)	8.8	0.007	1.8	0.035	5.0	0.035
PS(34:1)	5.2	0.001	1.0	0.033	5.2	0.033
PS(36:5)	6.4	0.045	8.0	0.384	0.8	0.384
PS(36:4)	9.5	0.001	1.9	0.011	4.9	0.011
PS(36:3)	9.6	0.001	2.8	0.020	3.5	0.020
PS(36:2)	9.5	0.003	2.4	0.050	4.0	0.050
PS(36:1)	10.8	0.007	2.7	0.010	4.1	0.010
PS(38:7)	16.5	0.038	11.4	0.274	1.4	0.274

PS(38:5)	20.9	0.009	3.0	0.016	6.9	0.016
PS(38:4)	13.2	0.020	8.2	0.313	1.6	0.313
PS(38:3)	18.4	0.068	1.6	0.001	11.3	0.001
PS(38:2)	10.3	0.030	3.2	0.072	3.3	0.072
PS(38:1)	12.6	0.040	3.4	0.095	3.7	0.095
PS(38:0)	9.9	0.082	3.2	0.002	3.1	0.002
PS(40:8)	7.8	0.001	3.8	0.194	2.1	0.194
PS(40:7)	13.1	0.002	4.5	0.095	2.9	0.095
PS(40:6)	21.4	0.015	8.7	0.199	2.4	0.199
PS(40:5)	20.0	0.003	7.7	0.226	2.6	0.226
PS(40:3)	5.2	0.046	0.7	0.068	6.9	0.068
PS(40:2)	14.1	0.016	1.9	0.016	7.5	0.016
PS(40:1)	7.2	0.023	1.9	0.021	3.8	0.021
PS(42:11)	8.3	0.048	1.9	0.049	4.4	0.049
PS(42:9)	11.6	0.007	4.8	0.031	2.4	0.031
PS(42:8)	5.5	0.011	1.8	0.011	3.1	0.011
PS(42:7)	41.8	0.000	13.7	0.103	3.0	0.103
PS(42:6)	23.0	0.030	2.5	0.008	9.4	0.008
PS(42:5)	15.1	0.074	2.1	0.031	7.3	0.031
PS(44:12)	13.3	0.024	2.9	0.005	4.6	0.005
PS(44:10)	10.1	0.001	5.3	0.208	1.9	0.208
PS(44:8)	17.2	0.019	8.5	0.112	2.0	0.112
PS(44:7)	6.2	0.104	1.0	0.042	6.4	0.042
PS(44:5)	11.7	0.012	5.0	0.180	2.3	0.180
PS(44:4)	16.1	0.005	4.5	0.023	3.6	0.023
PS(44:3)	16.3	0.001	6.0	0.083	2.7	0.083
PS(44:2)	14.3	0.000	4.7	0.056	3.1	0.056

Table S3. The differences in the hexosylceramide (HexCer), cholesteryl esters (CE) and diacylglycerol (DAG) lipidome of LD1 isolated from the livers of rats fed control (C), ethanol (E) or betaine-supplemented ethanol (EB) diet.

Lipid Species	C vs E		E vs EB		C vs EB	
	Fold change ↑	*p*-value	Fold change↓	*p*-value	Fold change ↑	*p*-value
HexCer-d18:1(20:0)	18.8	0.009236	5.1	0.0387	3.7	0.193
HexCer-d18:1(22:0)	15.0	0.001285	2.7	0.0224	5.5	0.034
CE(16:1)	12.4	0.015188	3.1	0.08527	4.0	0.039028
CE(16:0)	11.5	0.003855	3.8	0.023738	3.0	0.054119
CE(18:3)	13.8	0.004568	5.2	0.01803	2.6	0.093026
CE(18:2)	13.4	0.006543	5.9	0.025298	2.3	0.138044
CE(18:1)	14.5	0.005609	4.2	0.025987	3.4	0.035101
CE(18:0)	22.0	0.020207	4.0	0.06677	5.5	0.01337
CE(20:5)	7.5	0.001717	3.8	0.010198	2.0	0.195234
CE(20:4)	7.5	0.001717	3.8	0.010198	2.0	0.195235
CE(20:3)	14.8	0.005519	4.8	0.022255	3.1	0.070039
CE(22:6)	8.6	0.013031	3.6	0.032206	2.4	0.054285
DAG(16:0/16:1)	6.1	0.002937	0.9	0.432243	6.5	0.033
DAG(16:0/16:0)	8.0	0.000188	1.2	0.295031	6.5	0.033675
DAG(18:3/16:0)	12.0	0.000746	1.4	0.185356	8.4	0.040149
DAG(18:2/16:1)	23.6	0.000804	2.3	0.034355	10.3	0.03087
DAG(18:2/16:0)	13.9	0.000225	2.3	0.02018	6.0	0.038491
DAG(18:1/16:1)	22.5	0.000607	1.7	0.075445	13.6	0.009378
DAG(18:1/16:0)	12.7	0.000347	1.6	0.088075	8.1	0.015066
DAG(18:3/18:2)	16.5	0.005	3.5	0.031	4.7	0.050
DAG(18:3/18:1)	17.8	0.001	3.1	0.016	5.8	0.068
DAG(18:2/18:2)	19.8	0.010	5.5	0.035	3.6	0.082
DAG(16:0/20:4)	12.3	0.000	1.6	0.120	7.6	0.054

DAG(18:2/18:1)	19.7	0.004	3.4	0.030	5.7	0.047
DAG(16:0/20:3)	17.6	0.001	1.7	0.111	10.6	0.029
DAG(16:1/20:2)	20.2	0.029	4.1	0.090	4.9	0.012
DAG(18:2/18:0)	21.9	0.002	2.8	0.022	7.9	0.037
DAG(18:1/18:1)	16.1	0.002	2.2	0.045	7.4	0.020
DAG(18:1/18:0)	14.8	0.002	1.7	0.105	8.9	0.019
DAG(18:2/20:4)	20.2	0.001	2.5	0.028	8.0	0.067
DAG(16:0/22:6)	16.4	0.001	1.5	0.159	10.8	0.041
DAG(18:1/20:4)	18.6	0.000	2.2	0.047	8.5	0.066
DAG(18:2/20:3)	22.8	0.002	2.1	0.070	11.0	0.032
DAG(18:1/20:3)	11.7	0.001	1.0	0.482	12.0	0.060
DAG(16:0/22:4)	23.0	0.005	2.1	0.091	10.8	0.030
DAG(18:0/20:3)	23.4	0.001	1.9	0.098	12.1	0.070
DAG(18:1/20:2)	14.4	0.018	2.0	0.130	7.0	0.011
DAG(18:1/22:6)	27.0	0.000	2.7	0.015	9.9	0.047
DAG(18:2/22:5)	16.4	0.003	2.3	0.027	7.2	0.005
DAG(18:0/22:6)	30.3	0.001	1.9	0.104	15.8	0.064
DAG(18:1/22:5)	14.3	0.00205	1.9	0.114487	7.6	0.068519
DAG(18:2/22:4)	17.2	0.012797	1.0	0.492982	16.9	0.082295
DAG(18:1/22:4)	21.7	0.002175	2.1	0.079474	10.4	0.053206

Table S4. The differences in the triacylglycerol (TAG) lipidome of LD1 isolated from the livers of rats fed control (C), ethanol (E) or betaine-supplemented ethanol (EB) diet.

Lipid Species	C vs E		E vs EB		C vs EB	
	Fold change ↑	*p*-value	Fold change↓	*p*-value	Fold change ↑	*p*-value
TAG(48:0)	4.1	0.006	1.0	0.443	3.9	0.004
TAG(50:3)	9.6	0.004	1.2	0.358	8.2	0.027
TAG(50:2)	24.8	0.013	1.4	0.301	17.5	0.080
TAG(50:1)	14.3	0.010	1.8	0.141	7.8	0.052
TAG(50:0)	15.2	0.007	1.9	0.119	8.0	0.049
TAG(52:6)	15.7	0.002	1.8	0.106	8.8	0.043
TAG(52:5)	15.8	0.004	1.8	0.119	9.0	0.037
TAG(52:4)	34.1	0.001	1.9	0.067	18.1	0.028
TAG(52:2)	27.3	0.008	2.4	0.077	11.4	0.040
TAG(52:1)	19.6	0.013	1.6	0.219	12.2	0.049
TAG(52:0)	19.1	0.005	1.5	0.221	12.6	0.056
TAG(54:7)	11.9	0.016	1.1	0.411	10.4	0.045

Table S5. The differences in the cholesteryl esters (CE) lipidome of LD2 isolated from the livers of rats fed control (C), ethanol (E) or betaine-supplemented ethanol (EB) diet.

Lipid Species	C vs E		E vs EB		C vs EB	
	Fold change ↑	*p*-value	Fold change↓	*p*-value	Fold change ↑	*p*-value
CE(18:2)	2.0	0.0217	1.6	0.164	1.3	0.326
CE(18:1)	2.4	0.0143	1.5	0.176	1.6	0.178
CE(18:0)	1.9	0.0231	1.4	0.284	1.3	0.325
CE(19:0)	2.0	0.1138	5.5	0.046	0.4	0.055
CE(20:3)	2.3	0.0420	1.1	0.419	2.0	0.118
CE(20:2)	3.2	0.0255	1.8	0.171	1.8	0.136
CE(20:1)	2.2	0.0148	0.5	0.058	1.1	0.408
CE(20:0)	2.1	0.0691	0.1	0.042	0.3	0.094
CE(22:5)	2.8	0.0342	1.1	0.451	3.0	0.085

Table S6. The differences in the phosphatidylinositol (PI) lipidome of LD2 isolated from the livers of rats fed control (C), ethanol (E) or betaine-supplemented ethanol (EB) diet.

Lipid Species	C vs E		E vs EB		C vs EB	
	Fold change ↑	*p*-value	Fold change↓	*p*-value	Fold change ↑	*p*-value
PI(34:3)	1.6	0.021	1.7	0.440	0.9	0.071
PI(34:2)	1.4	0.025	1.9	0.223	0.7	0.037
PI(34:1)	1.4	0.004	1.9	0.218	0.8	0.031
PI(36:5)	1.3	0.136	1.9	0.195	0.7	0.046
PI(36:3)	1.7	0.013	1.9	0.390	0.9	0.031
PI(36:2)	1.9	0.001	1.9	0.482	1.0	0.035
PI(36:1)	1.8	0.006	1.9	0.419	0.9	0.043
PI(38:4)	1.8	0.025	1.6	0.334	1.2	0.107
PI(38:3)	1.8	0.055	3.1	0.105	0.6	0.018
PI(38:2)	1.6	0.005	2.0	0.258	0.8	0.023
PI(40:8)	0.5	0.082	1.4	0.040	0.4	0.327
PI(40:5)	4.6	0.003	2.6	0.155	1.7	0.012
PI(40:4)	1.6	0.192	5.4	0.048	0.3	0.067

Table S7. The differences in the phosphatidylserine (PS) and ePS lipidome of LD2 isolated from the livers of rats fed control (C), ethanol (E) or betaine-supplemented ethanol (EB) diet.

Lipid Species	C vs E		E vs EB		C vs EB	
	Fold change ↑	*p*-value	Fold change↓	*p*-value	Fold change ↑	*p*-value
PS(34:4)	5.7	0.049	3.1	0.071	1.9	0.249
PS(34:2)	2.1	0.022	1.7	0.142	1.2	0.386
PS(36:4)	2.2	0.052	4.5	0.043	0.5	0.094
PS(36:2)	2.3	0.017	2.7	0.021	0.9	0.415
PS(36:0)	7.1	0.021	49.4	0.020	0.1	0.094
PS(38:0)	0.9	0.426	2.5	0.212	0.4	0.047
PS(40:6)	0.2	0.013	0.3	0.160	0.6	0.237
PS(40:1)	2.3	0.012	2.2	0.043	1.0	0.481
PS(44:5)	3.4	0.025	3.6	0.031	0.9	0.449
PS(44:3)	1.7	0.015	1.4	0.194	1.2	0.358
PS(44:2)	2.0	0.035	1.4	0.188	1.4	0.271
ePS(36:3)	1.3	0.395	4.2	0.045	0.3	0.274
ePS(36:2)	2.7	0.022	2.6	0.068	1.1	0.466
ePS(38:4)	4.5	0.030	5.5	0.024	0.8	0.409
ePS(40:3)	1.2	0.374	4.4	0.043	0.3	0.114

Table S8. The differences in the phosphatidic acid (PA), Ceramides and cholesteryl esters (CE) lipidome of LD3 isolated from the livers of rats fed control (C), ethanol (E) or betaine-supplemented ethanol (EB) diet.

Lipid Species	C vs E		E vs EB		C vs EB	
	Fold change ↑	*p*-value	Fold change↓	*p*-value	Fold change ↑	*p*-value
PA(32:0)	4.6	0.021	3.8	0.048	1.2	0.354
PA(34:4)	8.5	0.003	2.0	0.039	4.4	0.003
PA(34:3)	0.0	0.074	1.9	0.280	0.0	0.001
PA(34:1)	5.6	0.042	3.2	0.079	1.8	0.109
PA(36:5)	2.5	0.018	3.3	0.049	0.8	0.390
PA(36:4)	5.9	0.029	1.9	0.167	3.2	0.039
PA(36:2)	16.6	0.007	5.6	0.013	3.0	0.178
PA(38:6)	0.0	0.028	0.8	0.383	0.0	0.051
PA(38:2)	8.5	0.044	6.5	0.078	1.3	0.337
PA(40:7)	2.1	0.162	3.9	0.024	0.5	0.310
Cer-d18:1(16:0)	2.4	0.016	1.6	0.205	1.5	0.287
Cer-d18:1(24:1)	2.3	0.001	1.3	0.073	1.7	0.005

Cer-d18:1(24:0)	2.7	0.000	2.1	0.005	1.3	0.107
CE(16:1)	4.1	0.001	1.0	0.421	4.2	0.000
CE(16:0)	4.3	0.003	1.2	0.244	3.7	0.000
CE(18:3)	5.4	0.008	2.1	0.044	2.6	0.002
CE(18:2)	4.3	0.019	1.7	0.134	2.5	0.004
CE(18:1)	5.0	0.002	1.5	0.061	3.3	0.000
CE(18:0)	8.7	0.000	1.8	0.034	4.9	0.018
CE(20:5)	2.7	0.103	1.1	0.410	2.3	0.023
CE(20:4)	2.7	0.103	1.1	0.410	2.3	0.023
CE(20:3)	8.1	0.016	1.9	0.113	4.3	0.000
CE(20:2)	10.4	0.010	2.6	0.053	3.9	0.000
CE(20:1)	7.8	0.001	2.5	0.002	3.1	0.002
CE(22:6)	5.2	0.001	2.0	0.011	2.6	0.000
CE(22:5)	6.0	0.005	0.8	0.264	7.4	0.002
CE(22:4)	17.0	0.008	2.0	0.085	8.6	0.000

Table S9. The differences in the phosphatidylcholine (PC) lipidome of LD3 isolated from the livers of rats fed control (C), ethanol (E) or betaine-supplemented ethanol (EB) diet.

Lipid Species	C vs E		E vs EB		C vs EB	
	Fold change ↑	*p*-value	Fold change↓	*p*-value	Fold change ↑	*p*-value
PC(28:1)	4.0	0.001	3.5	0.002	1.1	0.390
PC(30:1)	4.0	0.001	4.2	0.002	0.9	0.453
PC(30:0)	3.5	0.002	2.1	0.011	1.7	0.075
PC(32:2)	4.3	0.002	2.4	0.004	1.8	0.113
PC(32:1)	4.0	0.002	3.5	0.006	1.1	0.353
PC(32:0)	2.7	0.007	2.4	0.044	1.1	0.454
PC(34:2)	2.4	0.036	3.1	0.038	0.8	0.238
PC(34:1)	2.9	0.002	2.7	0.008	1.1	0.425
PC(34:0)	2.8	0.001	5.9	0.001	0.5	0.027
PC(36:3)	3.5	0.017	2.9	0.043	1.2	0.243
PC(36:2)	4.5	0.014	2.8	0.043	1.6	0.030
PC(36:1)	4.5	0.001	2.8	0.006	1.6	0.036
PC(36:0)	2.9	0.005	3.2	0.013	0.9	0.397
PC(38:5)	2.3	0.017	1.9	0.068	1.2	0.255
PC(38:3)	3.3	0.024	2.5	0.063	1.3	0.084
PC(38:2)	4.7	0.004	2.9	0.009	1.6	0.051
PC(38:1)	5.2	0.003	3.0	0.014	1.8	0.019
PC(38:0)	5.2	0.005	3.8	0.021	1.4	0.295
PC(40:8)	7.2	0.004	3.6	0.016	2.0	0.188
PC(40:7)	6.0	0.010	9.6	0.028	0.6	0.351
PC(40:6)	3.8	0.007	3.1	0.027	1.2	0.358
PC(40:5)	8.8	0.007	1.9	0.095	4.6	0.001
PC(40:2)	4.8	0.005	2.4	0.021	2.0	0.180
PC(42:11)	19.3	0.017	2.7	0.093	7.1	0.002
PC(42:10)	6.9	0.000	4.4	0.003	1.6	0.263
PC(42:9)	4.0	0.005	2.7	0.009	1.5	0.205
PC(42:8)	4.7	0.011	2.7	0.018	1.8	0.149
PC(42:7)	4.5	0.008	1.7	0.081	2.7	0.028
PC(44:7)	5.7	0.050	0.0	0.027	0.0	0.135
PC(44:4)	4.8	0.005	9.0	0.005	0.5	0.244

Table S10. The differences in the phosphatidylinositol (PI) lipidome of LD3 isolated from the livers of rats fed control (C), ethanol (E) or betaine-supplemented ethanol (EB) diet.

Lipid Species	C vs E		E vs EB		C vs EB	
	Fold change ↑	*p*-value	Fold change↓	*p*-value	Fold change ↑	*p*-value
PI(34:3)	6.4	0.027	2.0	0.143	3.2	0.025
PI(34:2)	5.5	0.006	2.1	0.051	2.6	0.044
PI(34:1)	5.2	0.003	2.0	0.041	2.6	0.024
PI(36:5)	5.3	0.036	1.8	0.172	3.0	0.007
PI(36:4)	5.5	0.018	2.3	0.077	2.4	0.005
PI(36:3)	5.5	0.004	1.9	0.044	2.9	0.007
PI(36:2)	6.3	0.001	1.6	0.059	3.9	0.009
PI(36:1)	6.5	0.000	1.9	0.007	3.5	0.002
PI(38:6)	7.7	0.025	2.3	0.100	3.4	0.006
PI(38:5)	7.3	0.022	2.0	0.107	3.6	0.003
PI(38:4)	3.4	0.042	1.5	0.186	2.2	0.021
PI(38:3)	5.3	0.001	2.1	0.007	2.6	0.007
PI(38:2)	9.5	0.001	2.4	0.015	3.9	0.062
PI(38:1)	58.6	0.009	1.8	0.224	32.9	0.117
PI(40:8)	5.4	0.005	1.0	0.488	5.3	0.069
PI(40:7)	10.1	0.057	1.6	0.273	6.3	0.011
PI(40:6)	11.8	0.016	3.3	0.053	3.6	0.083
PI(40:5)	21.3	0.040	1.8	0.227	11.5	0.028
PI(40:4)	10.3	0.020	2.7	0.093	3.8	0.028
PI(40:0)	13.7	0.070	1.4	0.350	9.8	0.047

Table S11. The differences in the phosphatidylethanolamine (PE) lipidome of LD3 isolated from the livers of rats fed control (C), ethanol (E) or betaine-supplemented ethanol (EB) diet.

Lipid Species	C vs E		E vs EB		C vs EB	
	Fold change ↑	*p*-value	Fold change↓	*p*-value	Fold change ↑	*p*-value
PE(28:1)	4.4	0.023	2.0	0.115	2.2	0.085
PE(28:0)	9.1	0.002	4.7	0.008	1.9	0.218
PE(30:1)	4.0	0.011	21.6	0.003	0.2	0.121
PE(30:0)	8.1	0.050	5.3	0.084	1.5	0.349
PE(32:1)	4.6	0.046	2.1	0.153	2.2	0.087
PE(34:4)	2.3	0.008	2.3	0.047	1.0	0.496
PE(34:2)	4.5	0.020	3.8	0.052	1.2	0.417
PE(34:1)	9.4	0.001	3.4	0.006	2.7	0.055
PE(34:0)	3.9	0.034	1.4	0.294	2.7	0.107
PE(36:3)	5.3	0.013	4.3	0.032	1.2	0.371
PE(36:2)	9.4	0.010	6.8	0.022	1.4	0.339
PE(36:1)	6.4	0.009	3.1	0.047	2.1	0.107
PE(38:2)	3.7	0.030	2.4	0.029	1.6	0.326
PE(38:1)	5.1	0.013	2.3	0.032	2.2	0.033
PE(40:8)	6.4	0.008	3.5	0.047	1.8	0.313
PE(40:5)	7.1	0.126	1.0	0.491	7.2	0.041
PE(40:3)	2.2	0.023	2.1	0.032	1.0	0.477
PE(42:10)	5.9	0.002	1.7	0.166	3.5	0.117
PE(42:9)	8.4	0.027	2.5	0.120	3.4	0.179
PE(42:3)	14.4	0.069	1.2	0.397	11.7	0.036
PE(42:2)	0.0	0.092	0.0	0.028	2.6	0.149
PE(44:11)	2.9	0.012	5.2	0.029	0.6	0.138
PE(44:8)	7.2	0.011	4.1	0.059	1.8	0.232
PE(44:6)	46.6	0.040	9.7	0.081	4.8	0.166
PE(44:5)	7.0	0.039	7.7	0.068	0.9	0.425
